# The Effect of Carbon Nanotubes on the Mechanical Properties of Wood Plastic Composites by Selective Laser Sintering

**DOI:** 10.3390/polym9120728

**Published:** 2017-12-18

**Authors:** Yunhe Zhang, Jing Fang, Jian Li, Yanling Guo, Qingwen Wang

**Affiliations:** 1 College of Mechanical and Electrical Engineering, Northeast Forestry University, Harbin 150040, China; fangjing_1217@163.com (J.F.); lijian499@163.com (J.L.); 2 College of Marterials and Energy, South China Agricultural University, Guangzhou 510642, China; qwwang@scau.edu.cn

**Keywords:** selective laser sintering (SLS), wood-plastic composites (WPCs), carbon nanotube (CNT), mechanical properties, binding mechanism

## Abstract

Wood-plastic composites (WPCs) made by selective laser sintering (SLS) approach of 3D printing offer many advantages over single polymer materials, such as low cost, sustainability, and better sintering accuracy. However, WPCs made via SLS are too weak to have widespread applications. In order to increase the mechanical properties of WPCs, a novel type of WPCs containing 0, 0.05, 0.1 and 0.15 wt % carbon nanotubes (CNT), 14 wt % wood fibers, 86 wt % polyether sulfone (PES) was manufactured via SLS. The experimental results showed that the addition of small amount of CNTs can significantly increase the mechanical properties of the wood/PES composite material. The tensile strength, bending strength, and elasticity modulus were 76.3%, 227.9%, and 128.7% higher with 0.1 wt % CNTs than those without CNTs. The mechanical properties of specimens first increased and then decreased with the addition of CNTs. The SEM results of the specimens’ fracture morphology indicate that the preferable bonding interfaces between wood flour grains and PES grains were achieved by adding CNTs to the composites. There are two reasons why the composites possessed superior mechanical properties: CNTs facilitate the laser sintering process of WPCs due to their thermal conductivities, and CNTs directly reinforce WPCs.

## 1. Introduction

Selective laser sintering (SLS) is an additive manufacturing technique. It can directly fabricate three-dimensional components via a computer-aided design model through selectively sintering powder-based layers materials. SLS possesses well process flexibility in regard to the raw materials that can be used [1], such as nylon, elastomer, and metal [2,3]. SLS has been one of the fastest growing additive manufacturing techniques used in many fields [4] because it has great potential to manufacture complex and low-volume parts more rapidly [5] and can also save more time and money compared with conventional manufacturing methods [6].

In recent years, there has been a growing need to develop new materials for 3D printing in order to lower costs [7]. Wood-plastic composites (WPCs), composed of cheap wood flour and waste polymer materials [8,9], are sustainable, so they have received attention from SLS researchers [10]. The use of WPCs in the SLS process offers some advantages over single polymer materials, such as better sintering accuracy [11], which is related to the low processing temperatures and the sintering mechanism that is different from neat polymers. However, the mechanical properties of WPCs made via SLS are poor such that they fail to find widespread applications.

Many efforts have been made to enhance the mechanical properties of SLS parts by means of incorporating reinforcements into the composite materials [12]. Gu et al. [13] found that direct laser-sintered Cu-based alloys can be reinforced by Ni particles and form a CuNi solid solution due to a coherent particle/matrix interface after solidification. Hon and Gill [14] fabricated silicon carbide/Polyamide composite materials through SLS and found that their tensile strength was lower than pure PA parts, but the composite parts’ stiffness was higher than pure PA parts. Some researchers found that C/C composite complex components with a high mechanical performance can be prepared by combining the 3D printing [15]. Furthermore, an increasing number of academic studies have focused on investigating the effects of nano-particles on polymer nanocomposites processed via SLS. Athreya et al. [16] found that the PA-12/carbon black nanocomposites made via SLS, compared with pure PA-12 SLS parts, revealed a better electrical conductivity but a lower bending modulus. This results from the poor dispersion of tiny carbon black particles and a weak interface between polymers and fillers. Yan et al. [17] found that adding 3 wt % nanosilica to PA-12 can improve the SLS parts’ thermal stability, tensile strength, and tensile modulus. Among the investigations of polymer nanocomposites processed via SLS, Yuan S. et al. found that the carbon nanotubes (CNT)/PA12 composite powders exhibited more thermal conduction and thermal absorption than pure PA12 powders [18]. CNTs as reinforcement were incorporated into polymeric composites to boost the laser sintering process and improve the thermal and mechanical performances of those composites.

Unfortunately, none of the reported studies have focused on the mechanical properties of sustainable wood plastic composites with CNTs (CNT/WPCs) processed via SLS. One study showed that CNTs increased the mechanical properties of rigid PVC/wood-flour composites by using a melted blending process [19]; however, the melted blending process does not have the same advantages as SLS. For example, the former requires the design and fabrication of high-cost part-specific tools and ties. There is little information about how CNTs act in CNT/WPCs processed via SLS.

This study focuses on the microstructure of CNT/WPCs parts manufactured via SLS, the influence of CNT content on mechanical properties, and the sintering mechanism of the specimens. The aim of the present study was therefore to find out the effects of the addition of CNTs on the mechanical properties of WPCs fabricated via SLS.

## 2. Materials and Methods

### 2.1. Raw Materials Characterization and Mixing Procedure

The wood powder used in the experiment was pine powder (45–90 μm) (Xingtai Kaifaqu Jinye Wood Powder Factory, Xingtai, China), and the polymer powder was polyether sulfone (PES, 60 μm) (Shanghai Tian Nian Materialas Technology Ltd., Shanghai, China). The chemical structural formula of PES is 
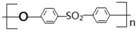
, and its average molecular weight is 35,000. Multi-walled CNTs were purchased from Chengdu Institute of Organic Chemisty (Chengdu, China) and had an average diameter of 50 nm and a length of 5–10 μm, and its purity level was greater than 95%.

**Figure 1:**
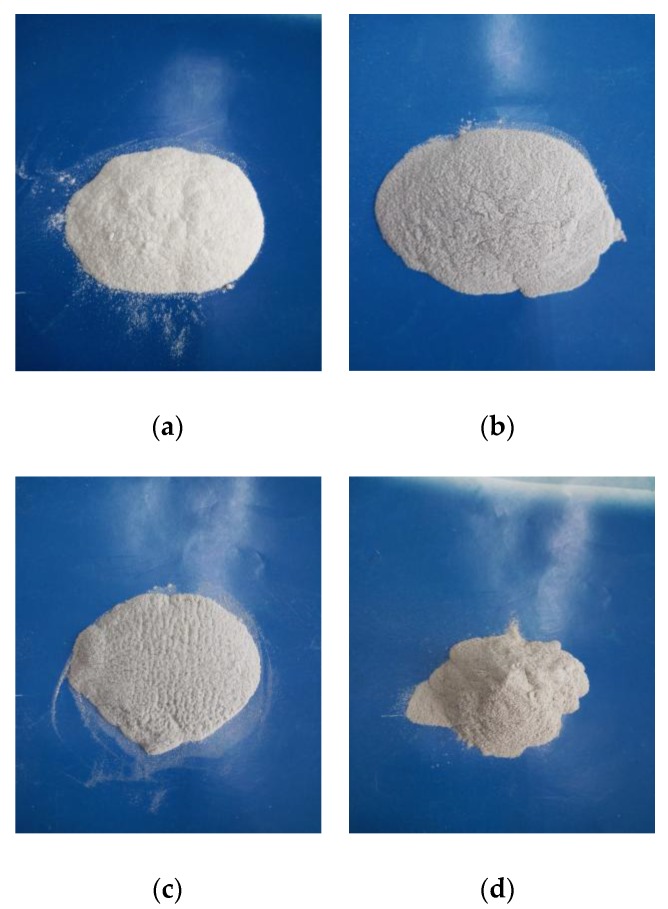
Optical images of the composite powder of wood flour, polyether sulfone (PES), and various amounts of carbon nanotubes (CNTs): (**a**) 0% CNT; (**b**) 0.05% CNT; (**c**) 0.1% CNT; and (**d**) 0.15% CNT.

The wood powder was over dried at 102 °C for 12 h until a constant mass was achieved to remove moisture before processing. The dried wood powder and PES with the mass fraction proportion of 1:6 were blended in a high-intensity mechanical mixer (type SHR-50A) (Lanzhou Zelin Chemistry Machinery Factory, Lanzhou, China) at room temperature for 2 min. CNTs were then mixed with the wood powder/PES composite powder by mechanical mixing. The CNT concentrations were respectively 0%, 0.05%, 0.1%, and 0.15% in weight. The composite powder without CNTs was white, and the composite powder, with the addition of CNTs, became grayer, as shown in Figure 1.

**Figure 2:**
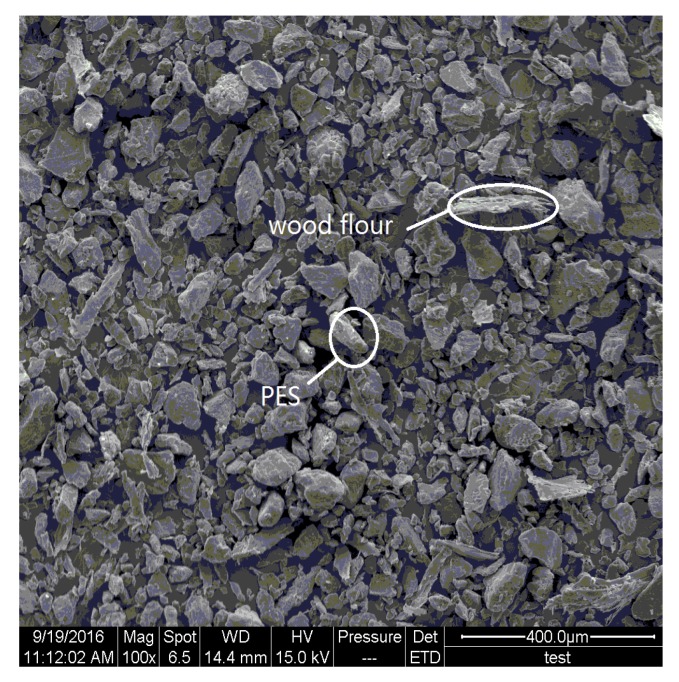
SEM image of the composite powder of wood flour, PES, and 0.1% CNTs.

Figure 2 shows an SEM image of the wood flour and the PES mixed at a ratio of 1:6 (by mass fraction) with 0.1% CNTs. As shown in Figure 2, the shape of the PES powder is subglobular and in various sizes. The wood powders present a rough surface with a shape of the fiber with aspect ratios from 2:1 to 8:1. The CNTs cannot be characterized in pictures with the low scale, because the size of the CNTs is very small and because the CNTs, which might cover the surface of the wood powder and PES particles, cannot be found easily.

The glass-transition temperature (*T*_g_) and melting temperature (*T*_m_) of the PES powder, the wood/PES and the CNT/wood/PES powder was obtained using the American Pyris Diamond differential scanning calorimeter (DSC) (Perkinelmer, Waltham, MA, USA). The heating rate was 10 °C·min/min from room temperature to 200 °C. DSC curves of the PES powder, the wood/PES powder, and the CNT/wood/PES powder were obtained.

### 2.2. Specimen Preparation and Mechanical Tests

The test specimens were sintered by a selective laser sintering machine (type AFS-360), product of Beijing Longyuan AFS Co., Ltd. (Beijing, China). Experimental parameters were set as follows: preheat temperature: 83 °C; internal power: 13 kw; external power: 3 kw.

Bending tests were carried out according to the standard of GB/T 9341-2008. Bending strength and elasticity modulus were tested with 23 specimens with four different CNT concentrations in the CNT/WPCs. A three-point bending method was applied. The dimensions of the specimens were 80 mm × 13 mm × 4 mm with a span length of 60 mm. Tensile tests were carried out according to the standard of GB/T 1040-2006. Tensile strength was tested with 36 specimens with four different CNT concentrations in the CNT/WPCs.

**Figure 3:**
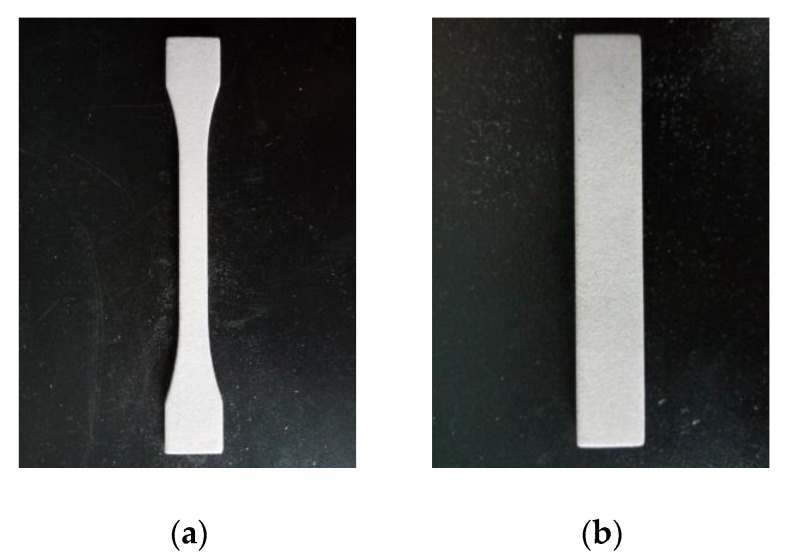
A photograph of the completed CNT/WPCs tensile test specimen (**a**) and the flexural test specimen by SLS (**b**).

The mechanical properties were tested with a universal mechanical machine (type CMT5504). The dimensions and shapes of the powder grains and the fracture morphology of the bending specimens were investigated with SEM (type FEI Quanta 200). Tensile test specimens were built successfully with good definition and uniform color without sintered lumps on the surface of specimens, as shown in Figure 3a,b, respectively.

## 3. Results and Discussion

### 3.1. Effect of CNT Content on the Mechanical Properties of CNT/WPCs Specimens via SLS

**Figure 4:**
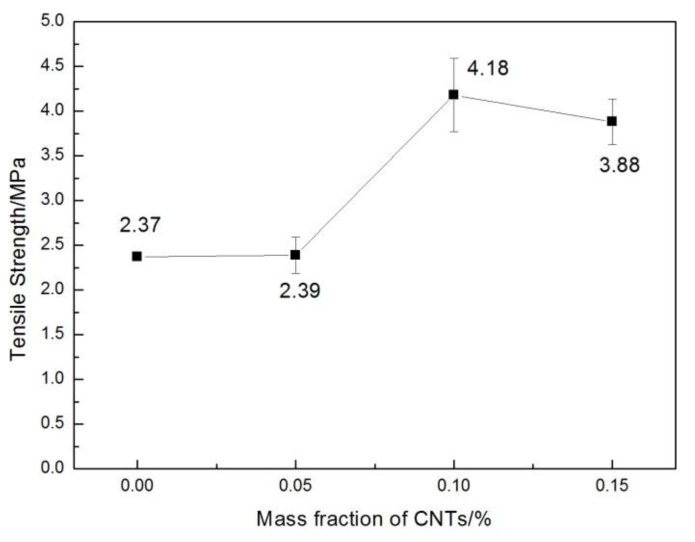
The effect of CNT content on the tensile strength of CNT/WPCs specimens by SLS.

**Figure 5:**
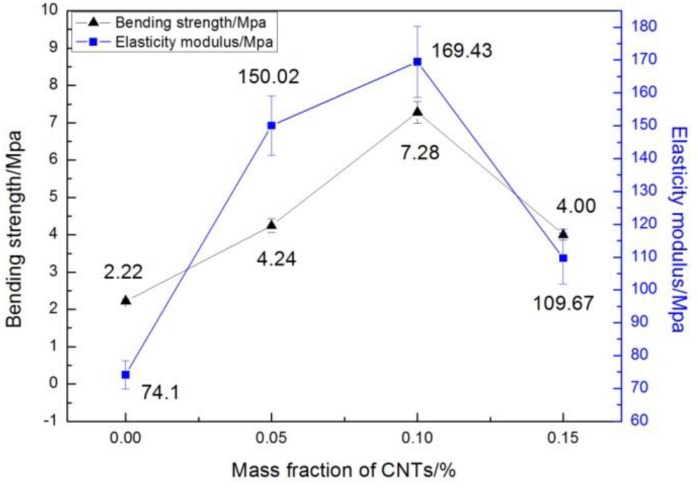
The effect of CNT content on the flexural strength and elasticity modulus of CNT/WPCs specimens by SLS.

The effect of CNT content on the tensile and flexural properties of the CNT/WPCs parts via SLS is shown in Figure 4 and Figure 5, respectively. CNTs greatly enhanced the tensile strength, flexural strength, and flexural modulus of the sintered components. Comparing with the pure WPCs specimens by SLS, the tensile strengths of 0.05%, 0.1%, and 0.15% CNT/WPCs sintered specimens increased by 0.8 ± 0.17%, 76.4 ± 9%, and 63.4 ± 5% respectively, the flexural strengths were enhanced by 91.0 ± 3.5%, 227.9 ± 5.54%, and 80.2 ± 4.54%, and the flexural modulus increased by 102.7 ± 5.3%, 128.4 ± 4.6%, and 48.0 ± 5.6% compared with their original values (ρ < 0.2, ANOVA) There was a significant increase in mechanical properties after only a small amount, 0.05–0.15 wt %, of CNTs were added to the WPCs. Bahereh T. Marouf, et al. [20] reported that the amount of loading necessary for an enhancement in crack-growth resistance is lower with silica nanoparticles compared with silica microparticles. This is evidence that silica nanoparticles, as opposed to silica microparticles, have a “nano-effect”. We can also infer that there a nano-effect of CNT was achieved here.

Although CNTs achieved an increase in the mechanical properties of WPCs via SLS, their strengths are still lower than CNTs processed via conventional fabrication methods, such as melt blending. It has been reported [18] that the bending strength of 5% CNT/wood/PVC via melt blending was 65 MPa, higher than the strength of the composites in this work. This is because CNT/WPCs via SLS as well as other composites via SLS are of many porosities.

Moreover, all mechanical properties tend to increase first and decrease later with the addition of CNTs. CNT/WPCs with a concentration of 0.1% possess the best mechanical properties, but at 0.15% these properties begin to decline. It has been reported that the much lower flexural modulus (over 20%) of the SLS composite parts compared with the pure PA-12 resulted from the agglomeration of 4 wt % carbon black fillers in the PA composite powders fabricated via mechanical mixing [5]. In the present study, a low mass fraction of only 0.15 wt % of CNTs caused a decrease in flexural modulus. This might be because there was more CNT agglomeration in the 0.15% CNT/WPCs compared with the 0.1% CNT/WPCs, which would be covered by a thinner layer of CNTs with less or no agglomeration.

### 3.2. Analysis of Fracture Morphology of CNT/WPCs via SLS

**Figure 6:**
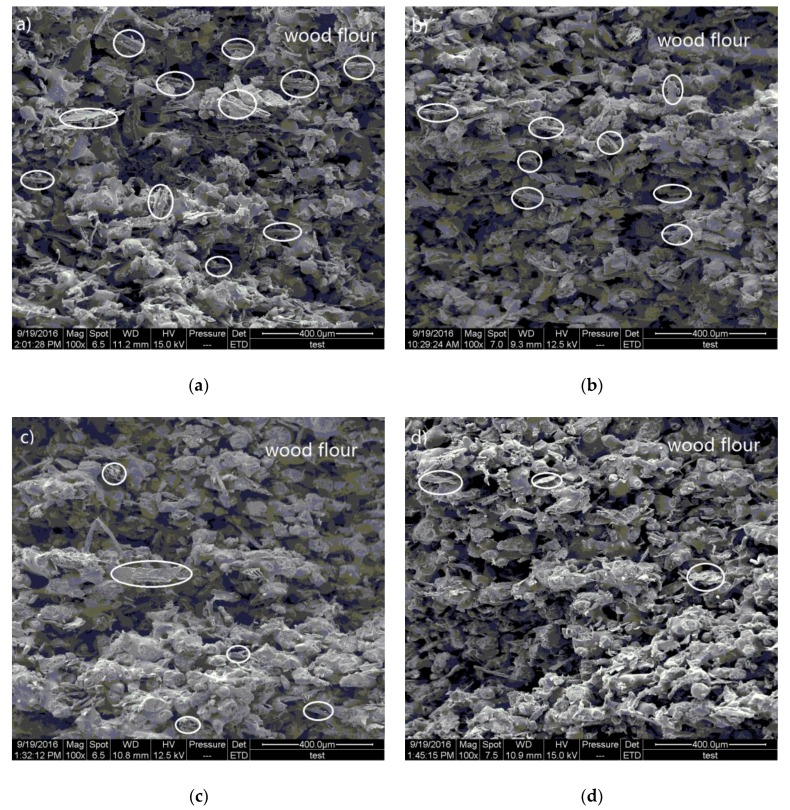
Lower magnification SEM images of fracture surface of specimens via SLS with CNT concentrations of (**a**) 0%; (**b**) 0.05%; (**c**) 0.1%; and (**d**) 0.15%.

In order to verify the morphological changes in the microstructure of the CNT/WPCs manufactured with CNTs, low- and high-magnification SEM images of fractured surface of the pure WPCs and CNT/WPCs parts via SLS were taken. Figure 6 shows the SEM images of the fracture at low magnification. It can be seen that the irregularly round PES particles partially melted and bonded to the surface of the wood fibers, which were joined by extensive con-continuous phase formation. There were also some pores on the surface that were formed under the moderate viscous flow of PES and the block of the wood fiber during sintering. These phenomena are similar to the previous study [21]. A visible difference between WPCs and various CNT/WPCs materials, however, can be observed. The pure WPCs sample presented relatively looser or more weakly linked powder particles and showed more bare wood fibers. CNT/WPCs showed a reduced number of bare wood fibers and an increasing amount of sintering necks. This indicates that low CNT content can facilitate the laser sintering of WPCs.

**Figure 7:**
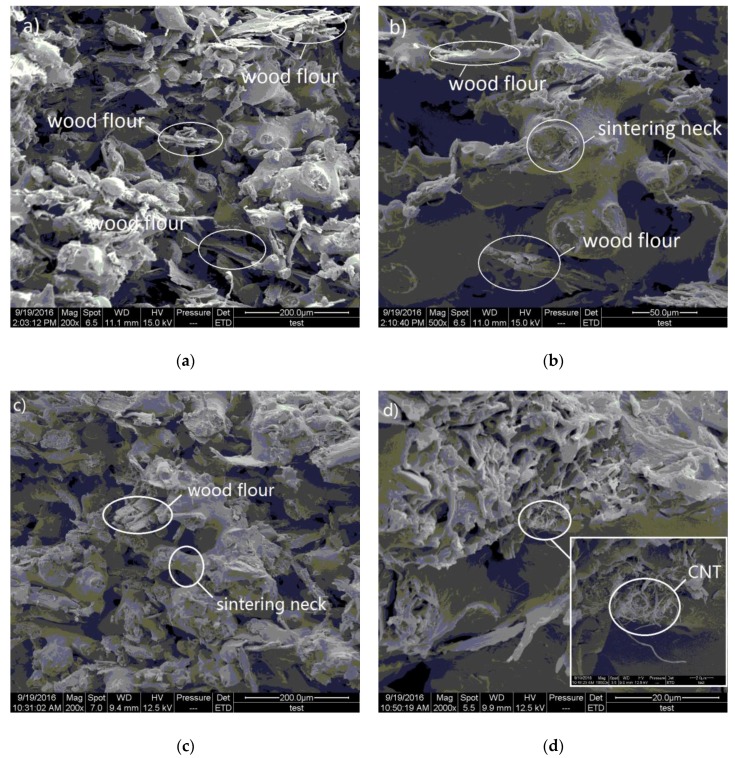

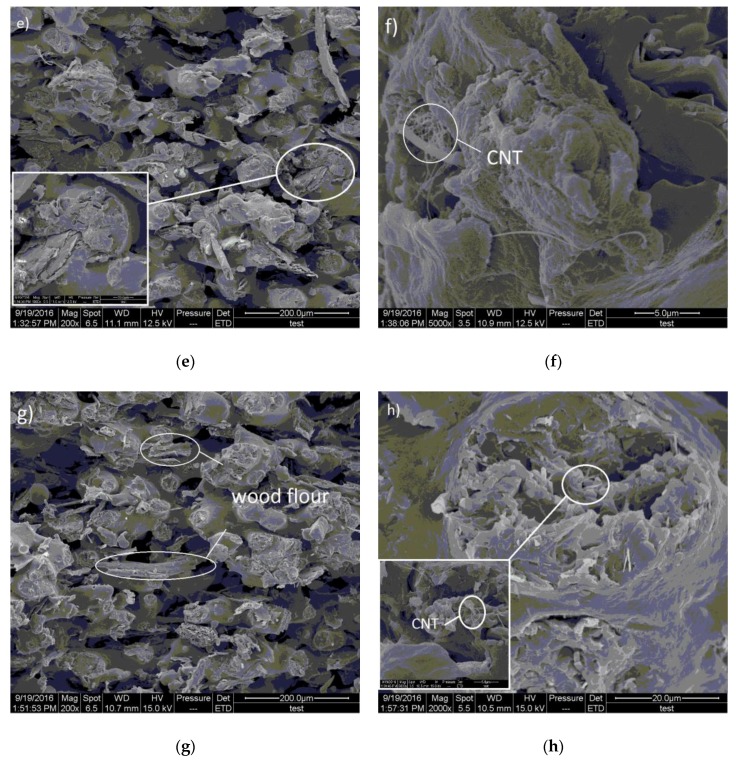
Higher magnification SEM images of fracture surfaces of specimens via SLS with CNT concentrations of (**a**,**b**) 0%; (**c**,**d**) 0.05%; (**e**,**f**) 0.1%; and (**g**,**h**) 0.15%.

Furthermore, higher magnification images of the four types of samples’ fractures offered more valuable information (Figure 7). Firstly, it can be seen that, with the increase in CNT content, the amount of bare wood fibers obviously decreased (Figure 7a,c,e,g), and some CNTs were located on the fracture surfaces of the CNT/WPCssintered parts (Figure 7d,f,h). There were also broken and unbroken sintering necks and a bonding area of two PES particles [22]. In Figure 7a,b, there are fewer and smaller sintering necks compared with Figure 7c–h. This reveals that the CNT/WPCs structures become denser as CNTs are added. When the amount of CNTs in the composite powder increased, PES particles melted and bonded together to a higher degree. In other words, the incorporation of CNTs caused more and bigger sintering necks. This explains well the variation of mechanical properties as CNT content changes (Figure 4 and Figure 5).

How CNTs work in sintering should be further analyzed. Jiang et al. [23] studied the effect of energy input, including laser power and part bed temperature, on the mechanical properties and microstructure of limestone/PA12 parts by SLS. It was found that the mechanical properties first improved as energy input increased, but decreased at higher energy inputs. This is because more energy inputs induce denser microstructures due to the melt flow rate of the PA12 polymer. Moreover, Bai et al. [24] found that, due to the greater thermal conductivity of PA12-CNTs, the laser heat was conducted wider and deeper in PA12-CNTs compared with PA12 alone. In the present study, CNTs also acted as a good thermal conductor between the laser beam and polymer powders of low thermal conductivity. Thus, the CNT/wood/PES powder can obtain more heat from the laser beam than wood/PES powder. Thus, energy input, such as laser power, must not be too high. This point is very important for the sintering of WPCs, because an overly high energy input can overheat wood fibers and burn them. CNTs might make the SLS parts of CNT/WPCs denser because of their high thermal conductivity.

**Figure 8:**
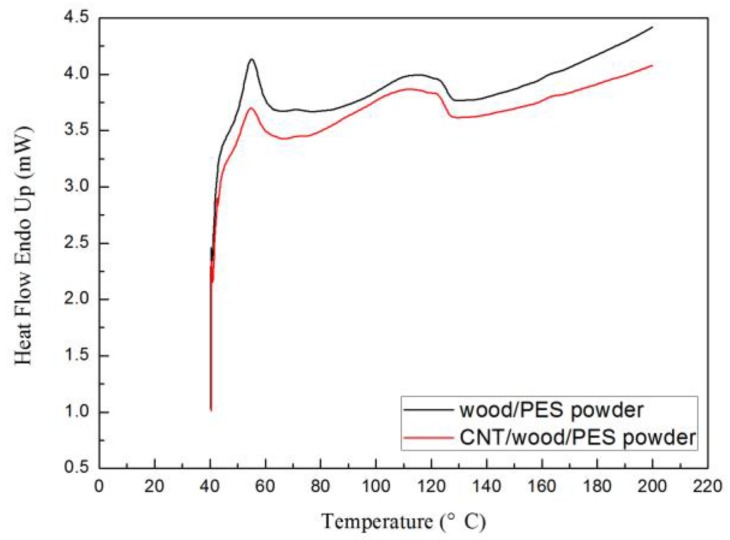
DSC curves of wood/PES powder and CNT/wood/PES powder.

Figure 8 shows the heating DSC curves of the wood/PES powder and the CNT/wood/PES powder (containing 0.1 wt % CNTs). It can be seen that there are few differences in the glass-transition temperature (*T*_g_) values of wood/PES powder and CNT/wood/PES powder, but the melting temperature (*T*_m_) of these types of powder changed from 113.3 to 110.6 °C when 0.1 wt % CNTs was added to the CNT/wood powder. Therefore, the high thermal conductivity of CNTs helps the CNT/wood/PES powder, as opposed to the wood/PES powder, to acquire more heat.

**Figure 9:**
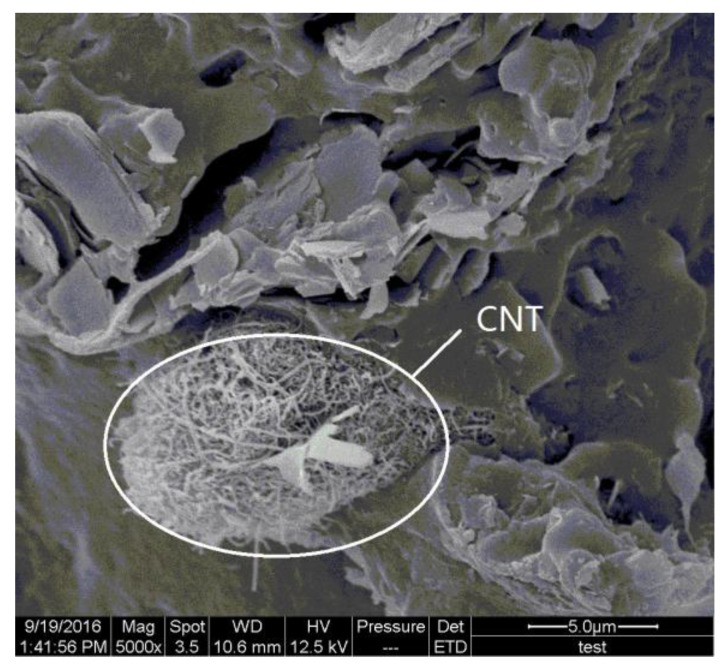
The agglomeration of CNTs in the 0.15% CNT/WPCs part by SLS.

The SEM images of mechanically fractured sections also provided valuable information for the analysis of fracture mechanisms (Figure 7). It is obvious that the main fracture mode of the pure WPCs is interfacial debonding between PES and wood fiber, and its secondary fracture mode is the fracture of the sintering neck, but this fracture is the CNT/WPCs’ dominant fracture mode. The former fractures are smooth and flat (Figure 7a), while the latter ones are rough (Figure 7c,e,g). A small amount of CNTs can be found in the fracture surface of the sintering necks (Figure 7d,f,h). This suggests that CNTs cause stronger interfaces between PES and wood fiber and that CNTs enhance the strength of polymer matrixes that include sintering necks. The CNT-coated layer, which covered the surface of the wood and polymer particles before sintering, had been well embedded within the melted polymer matrix during sintering. CNTs thus strengthen the polymer matrix and prevent the movement of molecular chains under loading—A conclusion similar to those arrived at elsewhere [18]. However, the mechanical strength showed a decreasing tendency at 0.15% CNTs in the WPCs. This might due to the CNT agglomeration caused by too much CNTs, as seen in Figure 9.

### 3.3. Schematic of the Sintering Mechanism during Laser Sintering of CNT/WPCs

**Figure 10:**
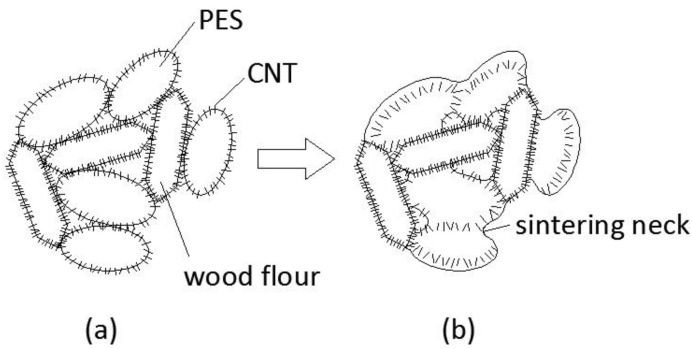
Sintering mechanism model of the CNT/WPCs (**a**) before sintering and (**b**) after sintering.

In order to clarify the effects of CNTs on the sintering procedures of CNT/WPCs, their sintering mechanism is schematically shown in Figure 10. The loose composite powder before sintering consists of PES particles, wood particles, and a layer of CNTs, which covered the surface of the PES and wood particles, as seen in Figure 10a. Here, the PES can be seen as a binding agent and the wood can be seen as a structural agent remaining solid throughout the process. The CNTs can be seen as a part of the binder PES, although CNTs, since they are smaller, do not melt like the wood particles do during sintering, and the CNTs are embedded into the melting polymer when the particles obtain the laser heat. Therefore, the CNT/WPCs material’s binding mechanism in SLS is liquid phase sintering-partial melting [22]. When the heat supplied to a powder particle was insufficient to melt the whole particle, only a shell at the grain border was molten and formed into a sintering neck. Only the rearrangement phase took place, so the sintering process was frozen at this stage, resulting in a porous green product, as shown in Figure 10b. CNT/WPCs, on the other hand, with more CNTs exhibited a strong tendency to form more sintering, which resulted in better mechanical properties. That is because CNTs with high thermal conductivity can transfer the heat from the laser beam to the PES particles. CNT/PES, compared with pure PES, can obtain more energy for the same length of time to obtain larger sintering necks. Meanwhile, PES can be reinforced by the CNTs embedded within it. This is verified by the CNTs pulled out in the center of the sintering neck fracture face, as shown in Figure 7.

### 3.4. Cost Analysis of CNT/WPCs

Natural fiber-like wood fiber is much cheaper than polymers such as PES. The market price of wood fiber and PES are $0.10 and $20/kg, respectively, so the price of the composite powder of 14% wood fiber and 86% PES in this study was $17.214/kg, with a decrease rate of 14%. Compared with pure polymer materials, this low cost is indeed one of the advantages of WPCs. The price of the cheapest CNT is $40/kg. CNTs in the present composites are 0.05–0.15 wt %, so the price of CNT/WPCs increases only by $0.02~0.06/kg. Thus, the price of CNT/WPCs remains the same as that of the WPCs (about $17/kg). 

On the basis of the above analysis, the cost of PES is the highest in the use of CNT/WPCs. Though this seems high, it is believed that the price of CNT/WPCs will fall dramatically if the content of PES is decreased if certain parameters are optimized and if the sizes of raw material particles of wood and polymer are well-adjusted in future research. 

## 4. Conclusions

A new type of low-cost, sustainable material—A mixture of low-content CNTs (0.05–0.15 wt %), wood fiber and PES powder—Was developed and used in SLS in this study. The effects of CNTs on the mechanical properties and microstructure of the CNT/WPCs materials were investigated. The experimental results indicated that tensile strength, bending strengths, and elasticity modulus of 0.10% CNT/WPCs were about 176%, 328%, and 229% higher than those without CNT-reinforced specimens. They increased first and then decreased with the addition of CNTs. Microstructure analysis showed that CNTs, as a good thermal conductor between the laser beam and the polymer powders, effectively enable the wood/polymer powders to obtain more heat from the laser beam such that the SLS parts of the CNT/WPCs were much denser than the WPCs after laser sintering. Secondly, CNTs on the surface of the composite powder grains were embedded into the polymer when the polymer partly melted during sintering, and they directly reinforced the strength of the composite parts. In addition, CNT agglomeration at 0.15% CNT/WPCs caused to lower the mechanical strength. This type of CNT/WPCs made via SLS with a higher strength, at low cost, and with greater sintering accuracy has widespread implications on additive manufacturing. These composites are expected to be used for many purposes, such as printing toys for children, printing molds for precision casting, and printing head sculptures in fine detail.
